# Intranasal fentanyl versus intravenous morphine in the emergency department treatment of severe painful sickle cell crises in children: Study protocol for a randomised controlled trial

**DOI:** 10.1186/1745-6215-13-74

**Published:** 2012-05-30

**Authors:** Michael Joseph Barrett, John Cronin, Adrian Murphy, Siobhan McCoy, John Hayden, SinéadNic an Fhailí, Tim Grant, Abel Wakai, Corrina McMahon, Sean Walsh, Ronan O’Sullivan

**Affiliations:** 1 Paediatric Emergency Research Unit (PERU), Emergency Department, Our Lady’s Children’s Hospital (OLCHC), Crumlin, Dublin 12, Ireland; 2 National Children’s Research Centre, OLCHC, Dublin 12, Ireland; 3 Department of Paediatrics, University College Dublin (UCD), Belfield, Dublin 4, Ireland; 4 CSTAR, Public Health and Population Science, Woodview House, UCD, Belfield, Dublin 4, Ireland; 5 Emergency Care Research Unit (ECRU), HRB Centre For Primary Care Research, Division of Population Health Sciences (PHS), Royal College of Surgeons in Ireland, 123 St. Stephen's Green, Dublin 2, Ireland; 6 Haematology Department, OLCHC, Dublin 12, Ireland

**Keywords:** Sickle cell disease, Paediatric, Pain, Analgesia, Intranasal, Fentanyl, Randomised Controlled Trial, Double blind

## Abstract

**Background:**

Children with sickle cell disease (SCD) frequently and unpredictably present to the emergency department (ED) with pain. The painful event is the hallmark acute clinical manifestation of SCD, characterised by sudden onset and is usually bony in origin. This study aims to establish if 1.5mcg/kg of intranasal fentanyl (INF; administered via a Mucosal Atomiser Device, MAD™) is non-inferior to intravenous morphine 0.1 mg/kg in severe SCD-associated pain.

**Methods/design:**

This study is a randomised,double-blind, double-dummy active control trial of children (weighing more than 10 kg) between 1 year and 21 years of age with severe painful sickle cell crisis. Severe pain is defined as rated seven or greater on a 0 to 10 age-appropriate numeric pain scale or equivalent. The trial will be conducted in a single tertiary urban paediatric ED in Dublin, Ireland. Each patient will receive a single active agent and a single placebo via the intravenous and intranasal routes. All clinical and research staff, patients and parents will be blinded to the treatment allocation. The primary endpoint is severity of pain scored at 10 min from administration of the study medications. Secondary endpoints include pain severity measured at 0, 5, 15, 20, 30, 60 and 120 min after the administration of analgesia, proportion of patients requiring rescue analgesia and incidence of adverse events. The trial ends at 120 min after the administration of the study drugs. A clinically meaningful difference in validated pain scores has been defined as 13 mm. Setting the permitted threshold to 50% of this limit (6 mm) and assuming both treatments are on average equal, a sample size of 30 patients (15 per group) will provide at least 80% power to demonstrate that INF is non-inferior to IV morphine with a level of significance of 0.05.

**Discussion:**

This clinical trial will inform of the role of INF 1.5mcg/kg via MAD in the acute treatment of severe painful sickle cell crisis in children in the ED setting.

**Trial registration:**

Current Controlled Trials ISRCTN67469672 and EudraCT no. 2011-005161-20

## Background

Children with sickle cell disease (SCD) frequently and unpredictably present to the emergency department (ED) [1]. In these children, pain is a common experience, beginning as early as 4 to 6 months of age [2]. These painful episodes (often referred to as a pain “crisis”) are the hallmark acute clinical manifestation of SCD, characterised by the sudden onset of pain. Although pain is a dominant feature in the medical lives of children with SCD, with approximately 70% of their hospitalisations after ED presentation being for uncontrolled pain [1-3], it is often under-recognised and undertreated [4-6]. Unrelieved pain does not only have negative consequences, such as missed days of school, restriction of other activities, and fear or mistrust of health care providers, but also can lead to amplified responses to subsequent pain experiences and sensitivity to pain later in life [2,7]. Furthermore, frequent painful episodes are associated with early mortality in patients with SCD [3,8].

Suboptimal acute pain management in SCD patients is associated with caregivers' preoccupation with the pathophysiological causes of vaso-occlusion and concern of creating opioid dependence [9], and a negative attitude toward patients with SCD in urban hospitals and EDs [5,9-11]. The truth regarding opiate addiction in patients with SCD is markedly less dramatic than conventional opinion would suggest. Prevalence estimates for opiate addiction among patients with SCD range from 0.5% to 8% compared to patients with other chronic pain syndromes with opiate addition rates of 3% to 16% [9,12,13]. Two putative biological mechanisms may explain the relatively low prevalence of opiate addiction among SCD patients. First, the allelic variants in the genes involving the opioid (UGT2B7, OPRM1, and ABCB1 genes) and non-opioid system (COMT gene) can alter the efficacy of morphine in SCD patients [14,15]. Second, the pharmacokinetics of morphine is altered in SCD patients, with clearance of morphine being 3–10 fold higher in SCD patients compared with healthy non-SCD patients [16].

Although treating acute painful sickle cell crisis (PSCC) with appropriate potent analgesia [intravenous (IV) and/or oral controlled-release morphine] is humane and reduces the length of hospitalisation in SCD patients with mild to moderate pain [17], the evidence base for acute ED pain management in children with PSCC is limited [18]. Alternative, quicker and pain-free methods of providing safe and effective analgesia include the intranasal (IN) route for the administration of opiates such as fentanyl, sufentanil and diamorphine [19-35]. The IN route is increasingly being used for the safe administration of analgesia in paediatric patients with painful conditions (e.g. burns, long bone fractures and post-operatively) [24,26,28,34,35]. In the emergency care setting, IN fentanyl is also currently used for managing pain in children in the pre-hospital setting [36,37]. Compared with other routes of drug administration, the IN route has unique advantages that may allow for more efficient use of resources, more rapid pain relief, and higher patient and provider satisfaction [38,39]. The highly vascularised nasal mucosa and the olfactory tissue in direct contact with the central nervous system permit rapid drug absorption, high bioavailability and onset of action comparable with IV drug administration [39]. Furthermore, IN drug administration is also relatively painless, inexpensive, convenient and easy to deliver with minimal training [39]. Fentanyl is a potent opioid (approximately 100 times more potent than morphine) with high bioavailability and a short duration of action (IN fentanyl plasma half-life of 1 h). When used in analgesic doses, fentanyl results in minimal sedation and little haemodynamic instability [40].

The primary aim of this study is to determine if 1.5mcg/kg of intranasal fentanyl via a Mucosal Atomiser Device (MAD™) is non-inferior to intravenous morphine in children presenting with severe pain associated with SCD.

## Methods/design

### Study aims

To determine whether 1.5mcg/kg IN fentanyl is non-inferior to 0.1 mg/kg IV morphine in the management of severe pain in children with PSCC.

### Study design and setting

This is a randomised, double-blind, double-dummy, active control clinical trial. It will be conducted at a single tertiary urban paediatric ED (Our Lady’s Children’s Hospital Crumlin (OLCHC)) in Dublin, Ireland.

### Subject selection

Children satisfying the screening inclusion and exclusion criteria, and the randomisation inclusion and exclusion criteria (Table 1), who present to the recruiting ED during the study period will be eligible for recruitment into the trial.

**Table 1:** Inclusion and exclusion criteria

*Inclusion criteria*:	
·	Ages 1 – 21 years
·	≥10 kg and ≤70 kg
·	Known sickle cell disease presenting with severe pain
·	Written informed consent, ideally from both parents (and assent, where appropriate), obtained prior to painful crisis (for example, in Haematology clinic)
·	Verbal consent (and assent, where appropriate) obtained at the time of the painful crisis in the ED
·	Hospital admission required for painful crisis
*Exclusion Criteria:*	
·	Patient has received parenteral narcotic analgesic within 4 hours of ED presentation
·	Oxygen saturations below 95% on initial assessment
·	Altered conscious state as defined by a Glasgow Coma score less than 15
·	Contraindications to fentanyl/morphine usage
·	Inability to secure IV access
·	Patient has participated in another clinical trial involving an Investigation Medicinal Product (IMP) within 4 weeks of dosing, or is currently enrolled in another clinical trial involving an IMP, or has been previously enrolled in this trial
·	Patients who have any condition that would make him/her, in the opinion of the Investigator or Sponsor, unsuitable for the study, or who are, in the opinion of the Investigator, not likely to complete the study for any reason
·	Blocked or traumatised nose

### Definition of painful crisis

An episode of severe pain is defined as the occurrence of pain in the extremities, back, abdomen or chest due to sickle-cell disease that is rated 7 or greater on a 0–10 numeric pain scale or equivalent [8].

### Inclusion and exclusion criteria

Inclusion and exclusion criteria are presented in Table 1.

### Patient randomisation

Patients who fulfil the randomisation eligibility criteria and whose parents provide informed consent will be randomised to receive intranasal fentanyl and intravenous placebo or intranasal placebo or intravenous morphine. The randomisation process was designed by CSTAR (Health Research Board Centre for Support and Training in Analysis and Research). Trial randomisation codes will be generated by CSTAR. The randomisation process provides a random allocation of 30 subjects between the two treatment groups. Randomisation will be on an individual basis using a computer-generated block randomisation. The randomisation is not stratified.

### Randomisation treatments

All study drugs will be packaged in blinded trial packs by a clinical trial pharmacist who is blinded to interventions and outcomes. Both fentanyl and morphine are controlled drugs. The Misuse of Drugs Acts, 1977 and 1984, and the Misuse of Drugs Regulations, 1988, 1993 and 2007, determine the conditions of production, possession, supply, importation and exportation of controlled drugs. Standard operating procedures (SOPs) are developed for every stage of a controlled drug’s journey from procurement (ordering, receipt and transport), safe storage, supply, administration, destruction and guidance for dealing with an incident. SOPs will be accessible to staff at all times.

#### Fentanyl and matched-placebo

Fentanyl citrate 50 μg/ml (Sublimaze, Marketing Authorisation No. PA 0748/044/001) is manufactured by Janssen Cilag, Ltd., and is an authorised product in Ireland. However, fentanyl is licensed for usage for paediatric pain via the intravenous route but not via the intranasal route. The matched size and shape of the placebo ampoule is necessary to maintain blinding of intervention by the investigators. The matched placebo for intranasal fentanyl is a 2-ml water for injection BP glass ampoule by Hamelyn Pharmaceuticals. The product is licensed in the UK for dissolving and diluting drug substances (reference no. PL 01502/0003R).

#### Morphine and matchedplacebo

Morphine sulphate 10 mg/ml BP is manufactured by Antigen Pharmaceuticals and authorised in Ireland (Marketing Authorisation No.PA 73/20/1). The matched placebo for intravenous morphine is a 1-ml water for injection glass ampoule by Roche Pharmaceuticals. It is an authorised product in Ireland for dilution of Clonazepam ampoules.

#### Overdosage considerations

In the considered event of an overdosage of opioid the approach of risk mitigation is as follows:

1. Trial pack manufacture Standard Operating Procedures;

2. Dispensing Standard Operating Procedures;

3. Monitoring of patient vital signs and clinical signs of opiate toxicity;

4. ED Staff training; and

5. Consideration of antidotal/reversal treatment (Naloxone is a standard stocked drug in the ED).

### Study procedure

#### Dose and administration schedule

**Figure 1:**
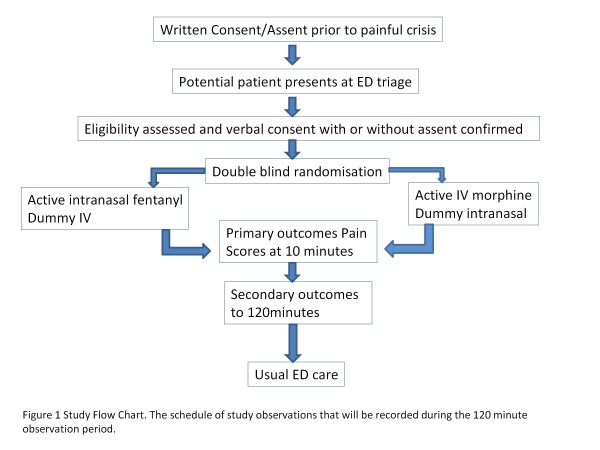
**Study flow chart.** The schedule of study observations that will be recorded during the 120-min observation period.

The study drugs will be administered at time 0 as outlined in the study flow chart (Figure 1). The patient’s age-appropriate pain score will be recorded at time points 0, 5, 10, 15, 20, 30, 60 and 120 min by a single investigator on the patient’s case report form. After the end of the 120 min the study will be terminated. The Faces, Legs, Activity, Cry, Consolability (FLACC) scale and Manchester Pain Ruler will be used as age-appropriate pain scales for pre-verbal/early verbal children and older verbal children respectively.

### Outcome measures

The *primary outcome measure* is severity of pain as measured using a validated pain score at 10 min post-analgesia.

The secondary outcome measures are:

· Severity of pain as measured by pain scores at 0, 5, 15, 20, 30, 60 and 120 minutes after the administration of analgesia;

· The proportion of patients requiring rescue opioid analgesia; and

· Adverse events within the 120 minutes study period.

### Sample size, power and statistical methods

The purpose of this study is to establish non-inferiority in pain relief for IN fentanyl as compared to IV morphine in the ED treatment of severe painful sickle cell crises in children. The study is a repeated measures study, measuring pain scores before and after the treatment. A detailed statistical analysis plan has been developed in association with CSTAR. From a previous study it is estimated that the reduction in pain will be 26 mm on a visual analogue score (VAS) with a standard deviation of 6 mm [27]. As a non-inferiority study, a threshold of how different the two treatments are has to be established, generally from clinical data. A clinically meaningful difference in VAS scores has been defined as 13 mm [41,42]. Setting the allowable threshold to 50% of this limit (6 mm) and assuming the two treatments are on average equal, a sample size of 30 patients (15 per group) will provide at least 80% power to demonstrate that INF is non-inferior to IV morphine with a level of significance of 0.05.

### Data analysis

Non-inferiority of the intranasal fentanyl treatment will be established based on a repeated-measure analysis of variance (ANOVA) with time of measurement (before or after treatment) as the within-subjects factor and treatment as the between-subjects factor. Data will be analysed on an intention-to-treat and a per protocol basis.

## Discussion

### Ethical considerations

This study is to be performed in accordance with the Good Clinical Practice (GCP) Guidelines, the EU CT Directive 2001/20.EC, GCP Commission Directive 2005/28/EC, the Declaration of Helsinki (2008) and all other local regulatory requirements. Risk analysis was carried out as part of protocol development. The study protocol was approved by the institutional Health Research Ethics Committee (HREC), OLCHC, Dublin. Patients will be first screened and consented/assented in the Haematology Outpatient Department and upon arrival in the ED, and upon meeting the study eligibility criteria will be verbally re-consented/assented in the ED.

### Regulatory considerations

The Irish Medicines Board (IMB) is the competent authority for the review and approval of clinical trials with an investigational medicinal product in Ireland. The application process for a medicinal trial was successful after formal engagement with the IMB prior to application and during the application process. IMB approval for this trial was granted on 20 January 2012.

### Breaking of the study blind

#### During the study

If an adverse event is regarded as a potential serious unexpected suspected adverse reaction (SUSAR) by the sponsor, the treatment group to which the trial subject affected belongs is unblinded for that subject alone. The procedure will ensure that the identity of the IMP is only revealed as far as necessary (GCP Directive). All staff will have received training on all aspects of the trial protocol prior to commencement of the trial.

The principal investigator (PI) or authorised member of the team will have a written procedure for requesting randomisation codes for rapidly identifying a blinded IMP in an emergency. Breaking the blind of a trial subject will be at the discretion of the PI, when clinically indicated for the safety of the patient or in the event of a SUSAR. If the patient needs to be unblinded we will refer to the unblinding SOP for complete details of the procedures to be followed. The master randomisation codes will be kept by the clinical trial pharmacist and the PI. Unblinding will be performed by the senior clinician/pharmacist when the criteria for a serious adverse event (SAE)/SUSAR have been met, and there is a necessity for the PI or treating healthcare professional to know which treatment the patient is receiving to ensure that the patient receives appropriate urgent safety measures.

A 24-h contact number will be available in the circumstances when unblinding is required. The scenario will be communicated and when the unblinding criteria are met the unblinding will ensue. The PI will document the breaking of the code, and the reasons for doing so, in the site file and in the patient’s medical notes, and in accordance with the clinical trial protocol.

#### Following completion of the study

Study unblinding will only take place once the statistical analysis plan has been agreed upon by the trial team and the final database locked.

### Bias and confounding variables

In terms of selection bias, we feel that this study targets a patient population to whom this research ultimately will be clinically applicable and valuable. Every effort will be made to ensure that recruitment of participants occurs over all 24-h periods (including weekends) by having patients recruited by the ED physician treating the patient and consented at haematology outpatients in advance of a PSCC or as an inpatient in OLCHC in anticipation of their next crisis. We anticipate that the randomised, double-blinded, controlled design of this study will minimise the effect of confounding variables on our analysis.

### Safety reporting

All adverse events that occur during the study period observed by one of the clinical staff, or reported by the patient or parent/guardian spontaneously, or in response to a direct question, will be noted on the appropriate form [i.e. adverse event (AE), SAE or SUSAR form]. These forms are de-identified. The following procedures will take place depending on the type of event that has occurred.

### Adverse event (AE)

Each AE will be recorded by a member of the research team on an AE form. Adverse events will be classified on the form in terms of their severity, association with the study drug, expectedness and seriousness. They will be recorded on an adverse event log. The adverse events will be reported to the sponsor and the institutional HREC on a yearly basis as part of an annual safety report and at the end of the trial.

### Serious adverse event (SAE)

Each SAE will be recorded by a member of the research team on an SAE form. SAEs will be classified on the form in terms of their severity, relatedness to the study drug and expectedness. They will be recorded on aserious adverse event log. All SAEs will be reported on the SAE form within 24 h to the sponsor and the institutional HREC. The research team will ensure that follow-up information and a detailed written report are provided when available. An ongoing prospective study of children experiencing severe painful sickle cell crisis in our ED reveals that over 90% of these children are admitted as an inpatient for further management of their crisis. This, therefore, would not be truly indicative of an SAE in this cohort of patients and will not be classified as such.

### Suspected unexpected serious adverse drug reactions (SUSAR)

Each SUSAR will also require expedited reporting to the sponsor. This will occur as soon as possible, but no later than 24 h after a member of the research team has first knowledge of the minimum criteria for expedited reporting. In each case, relevant follow-up information will be sought and a detailed, written report completed as soon as possible. The sponsor has responsibility to ensure all relevant and available information is forwarded to the competent authority (Irish Medicines Board) and the appropriate health ethics committee (HREC, OLCHC). For fatal or life-threatening events this will be done as soon as possible and not later than 7 days after the sponsor becomes aware of the event. Additional relevant information will be sent within 8 days of the first report. This will be sent no later than an additional 15 calendar days. For AEs that are not fatal or life-threatening, the sponsor will ensure that a SUSAR is reported as soon as possible and in any event not later than 15 days after the sponsor is first aware of the event.

The parents of participants will be provided with 24-h contact details of a study representative if they have concerns about any component of the study or their child’s condition. We will also report to the hospital (OLCHC) Risk Management Team and the Drugs Advisory Committee of the study site.

## Trial status

Recruitment commenced in April 2012 for the 30 patients needed for the study. It is anticipated patient recruitment will be completed by early 2013.

## Abbreviations

PSCC, Painful sickle cell crisis; ED, Emergency department; SCD, Sickle cell disease; IN, Intranasal; OLCHC, Our Lady’s Children’s Hospital Crumlin; MAD, Mucosal atomiser device; CSTAR, Centre for Support and Training in Analysis and Research (HRB); SOP, Standard operating procedure; IV, Intravenous; VAS, Visual analogue score; AE, Adverse event; SAE, Serious adverse event; SUSAR, Suspected unexpected serious adverse drug reaction; IMB, Irish Medicines Board; PI, Principal investigator; GCP, Good clinical practice.

## Competing interests

The authors declare that they have no competing interests.

## Authors’ contributions

ROS conceived the study. MB, JC, AM, SMC, JH, SNF, AW, CMcM, SW and ROS each made substantial contributions to the study design; were involved in drafting the manuscript and revising it critically for intellectual content; and gave final approval of the version to be published. TG provided the statistical support. This forms part of MB’s MD thesis registered with University College Dublin, Ireland. All authors read andapproved the final manuscript.
